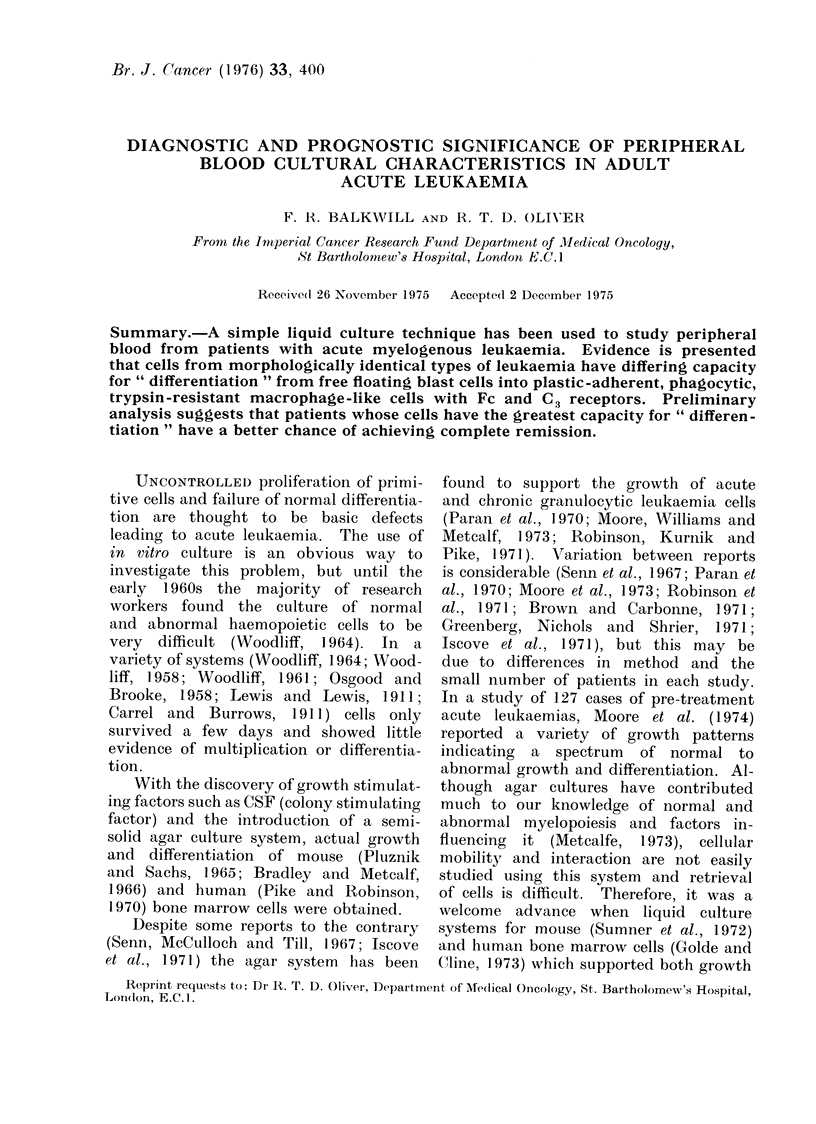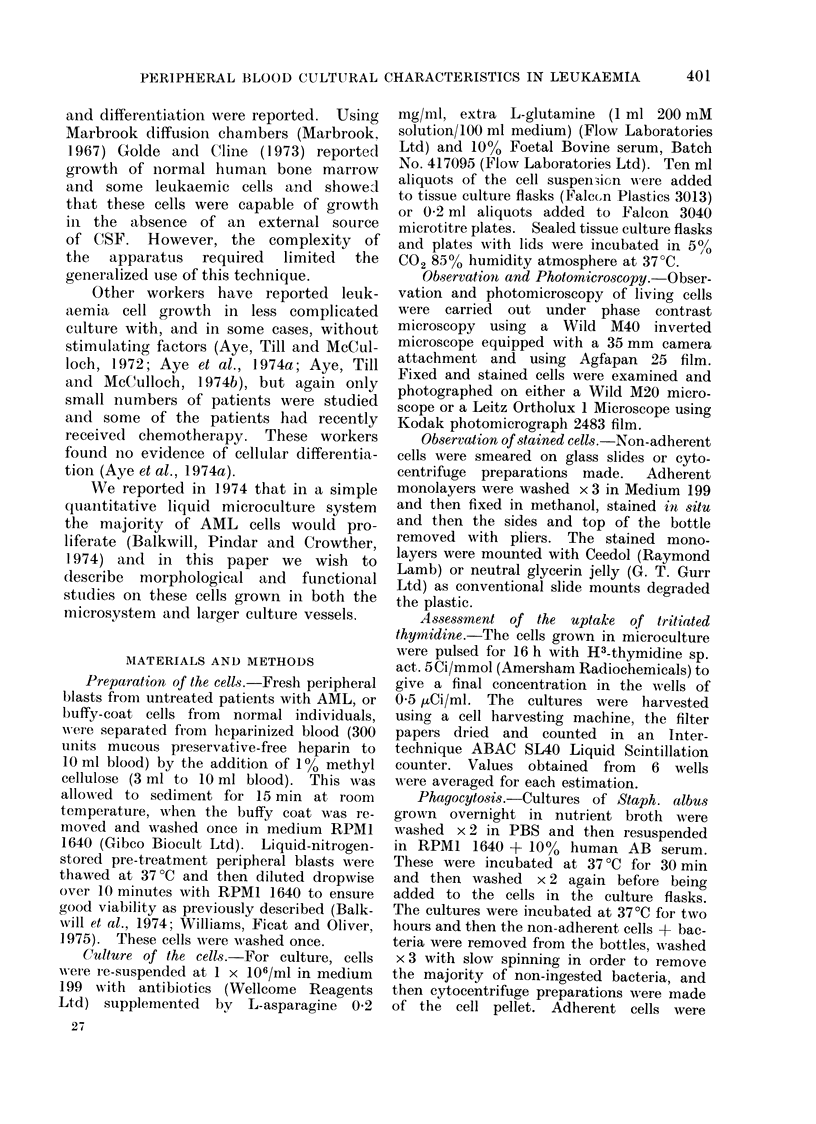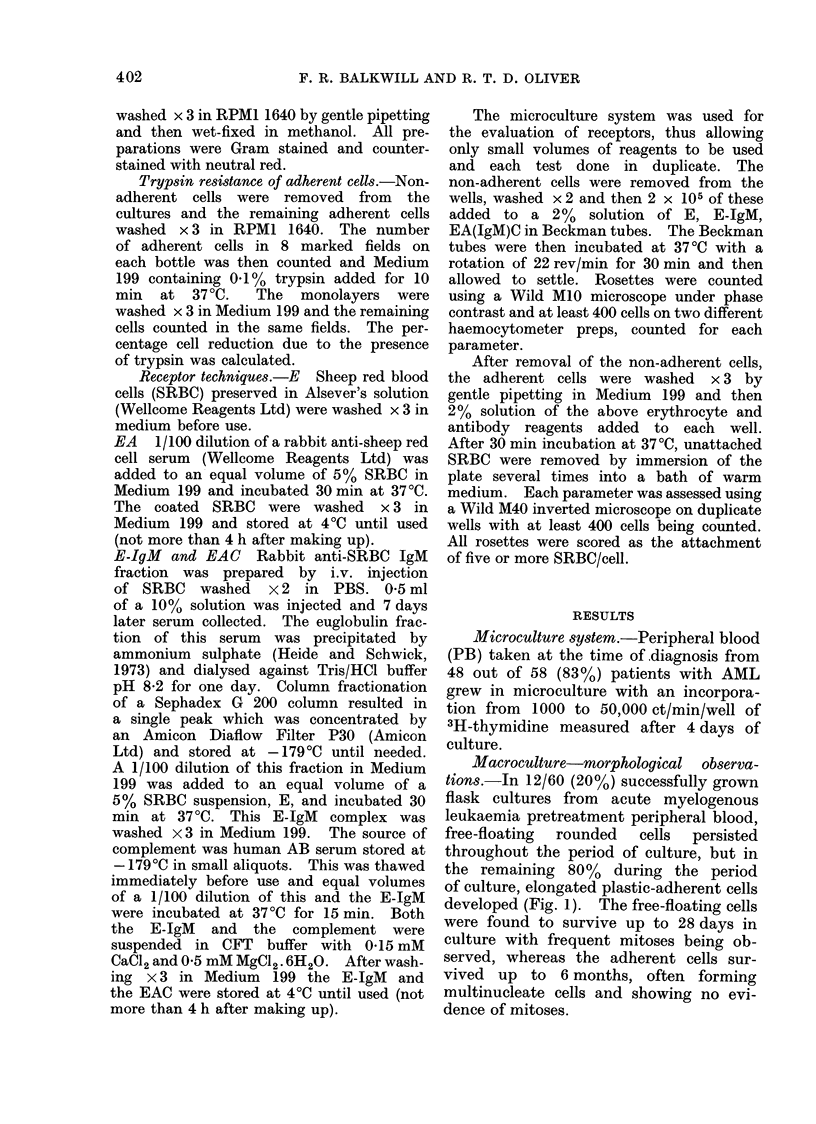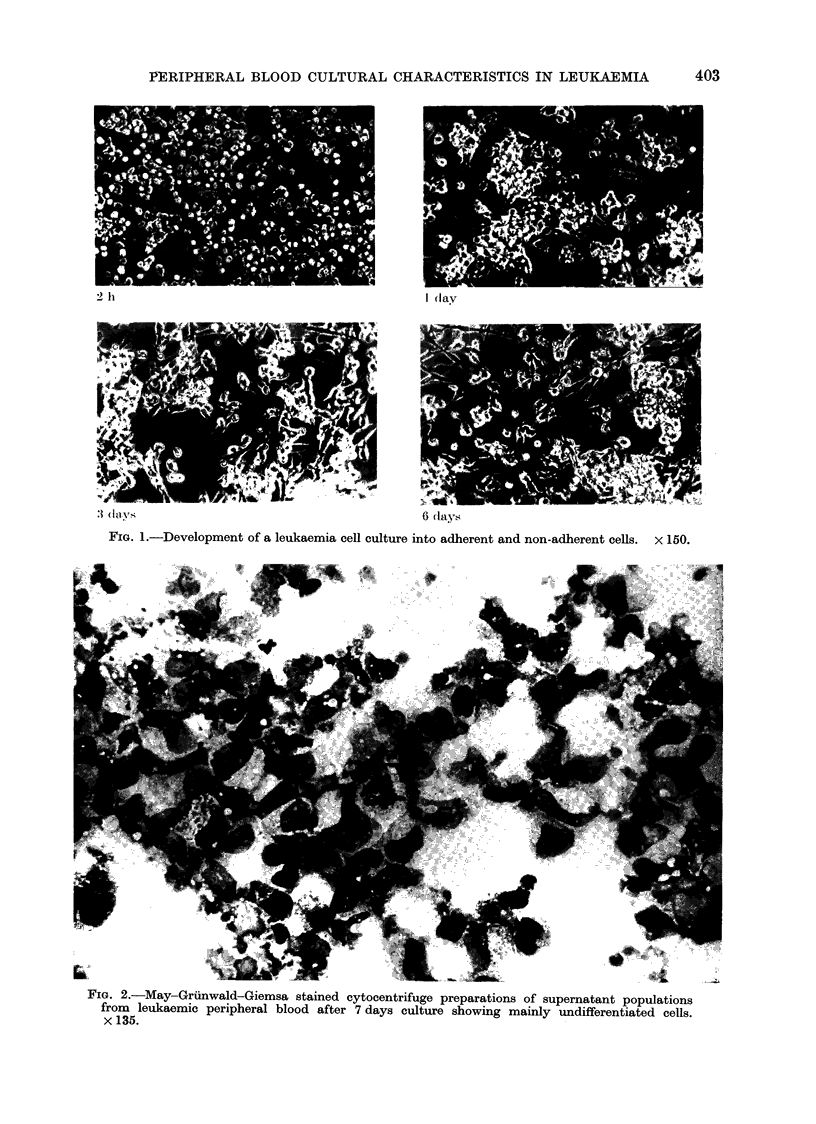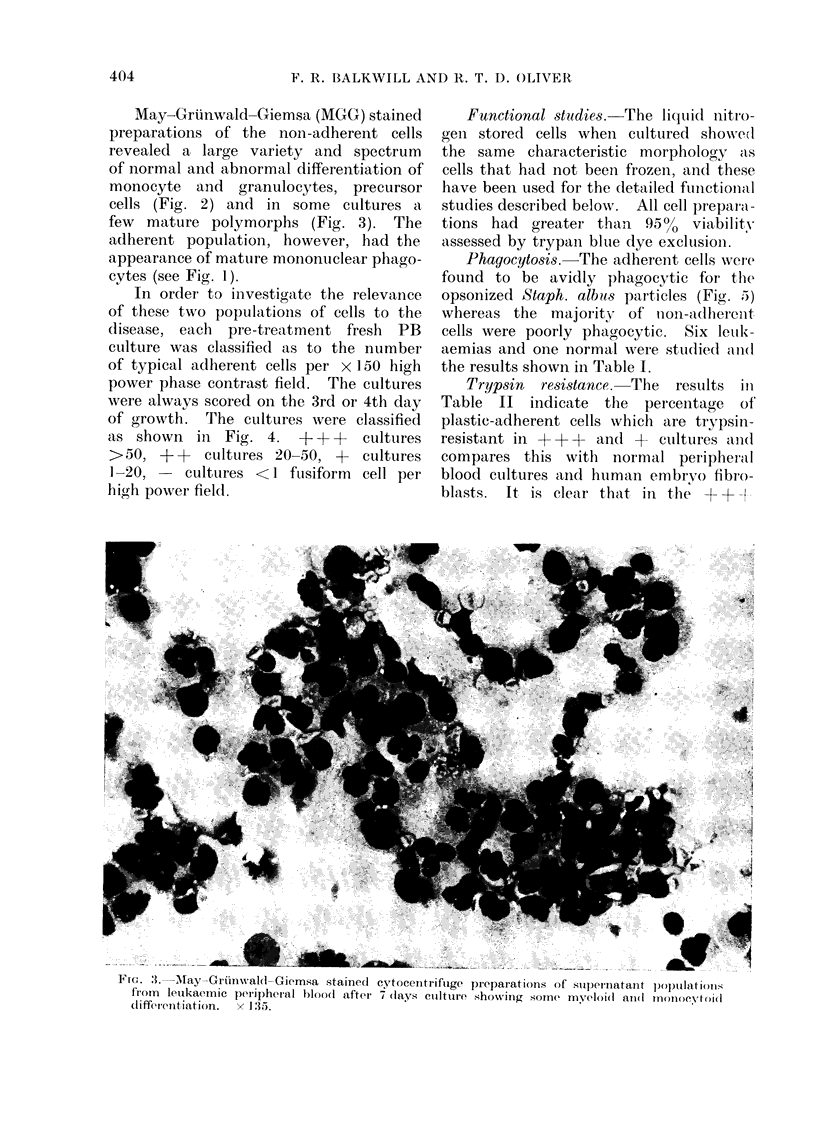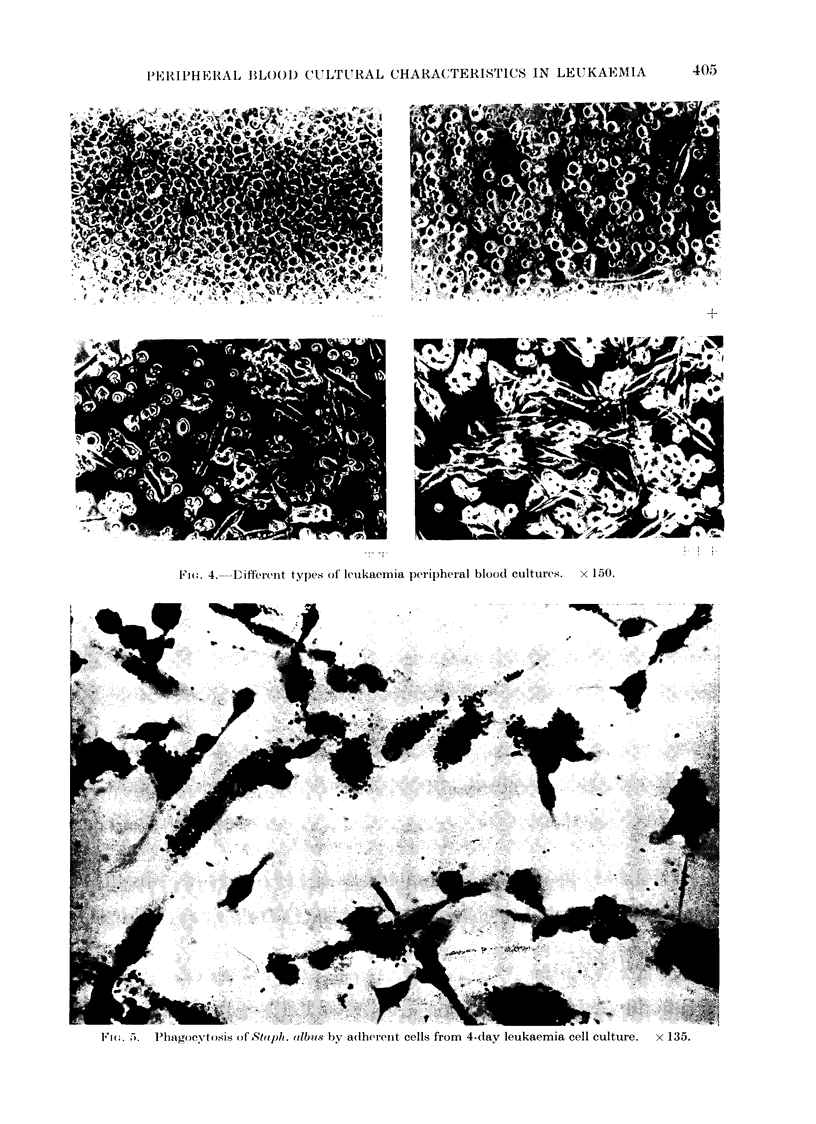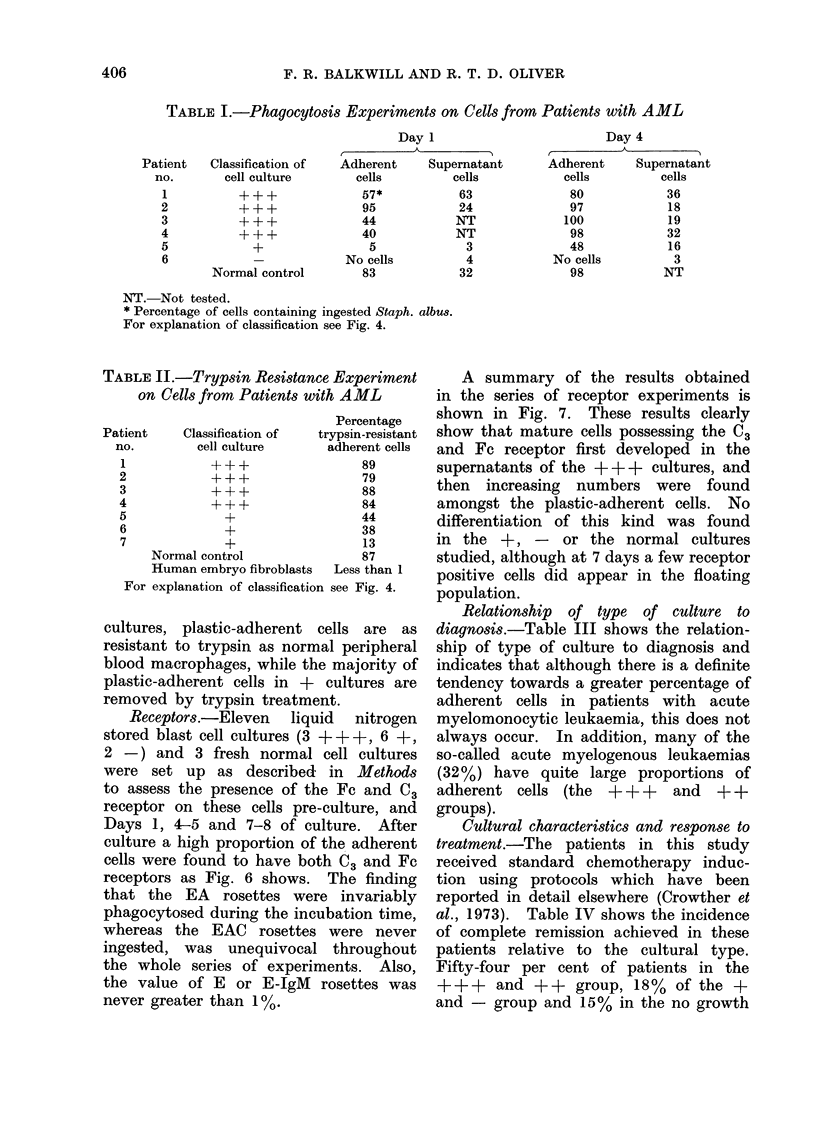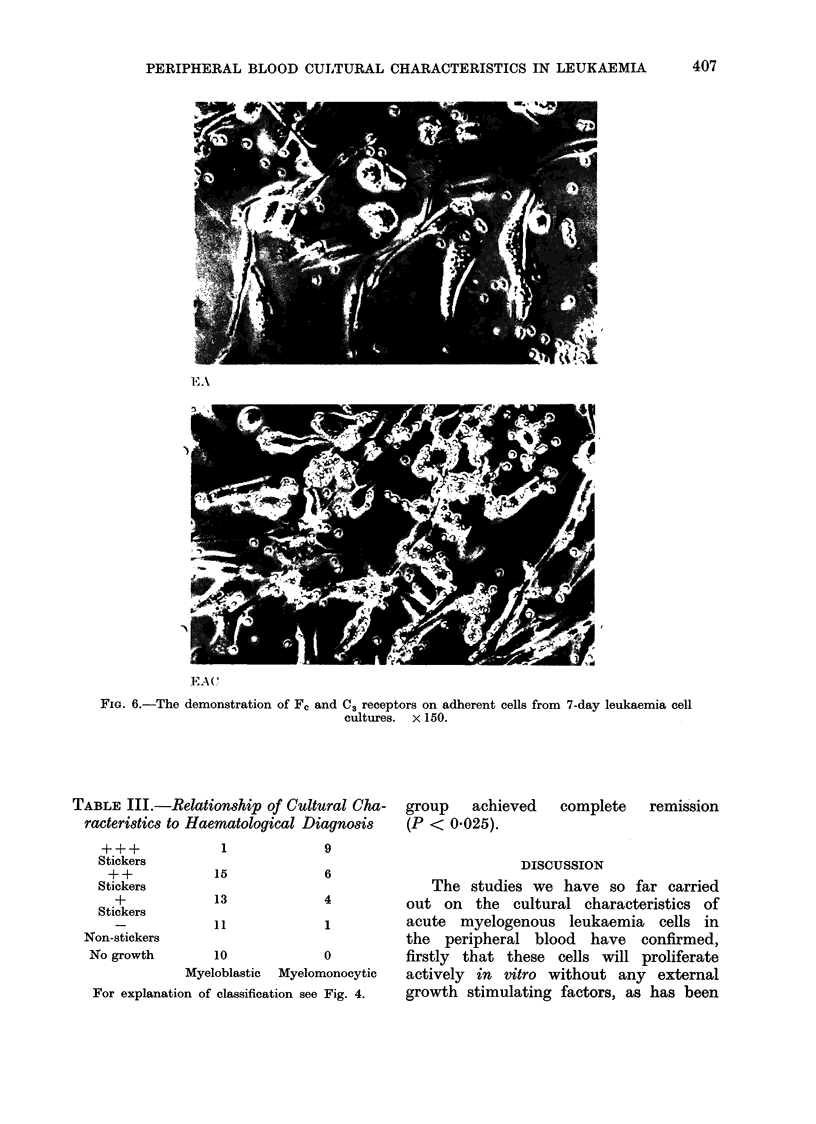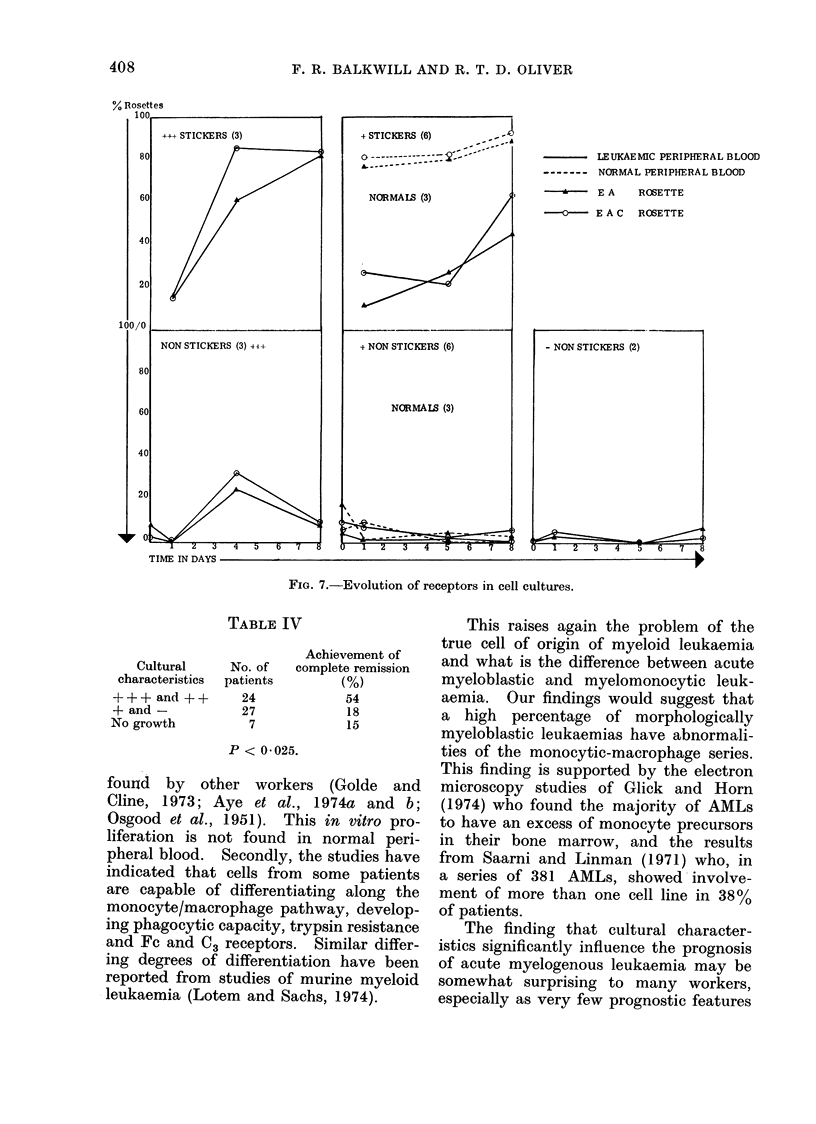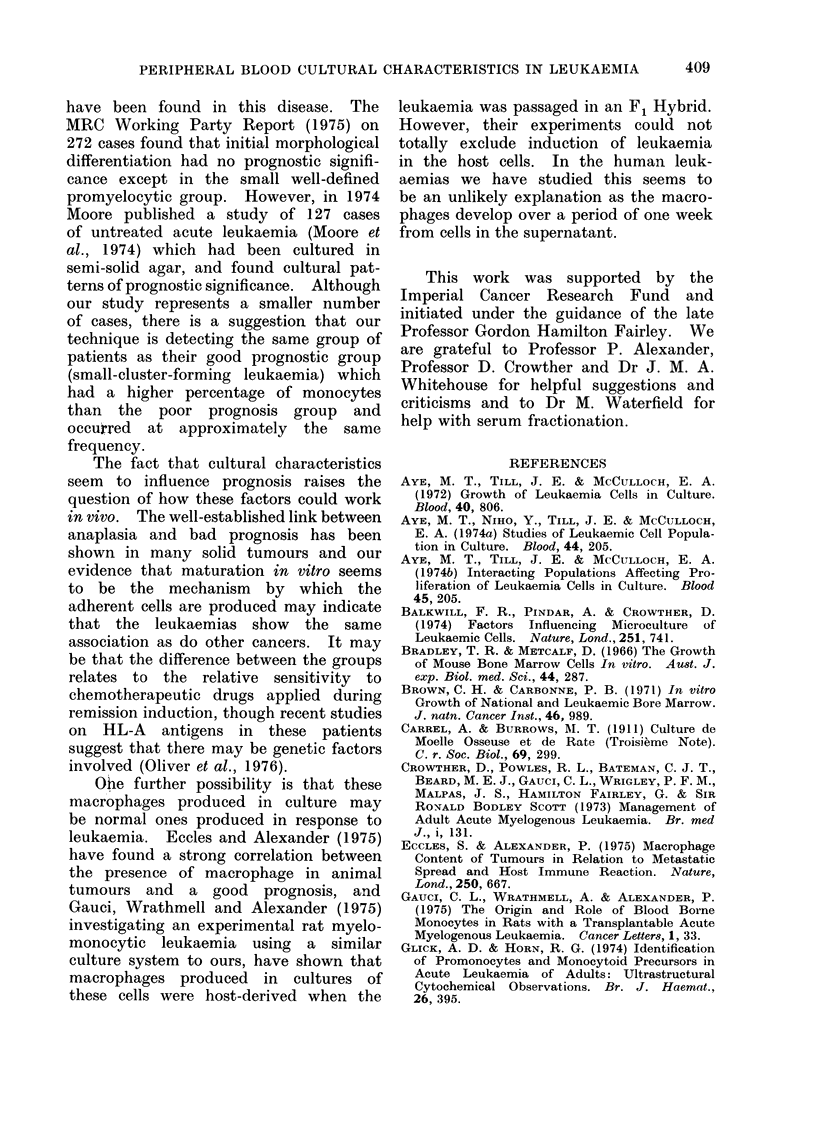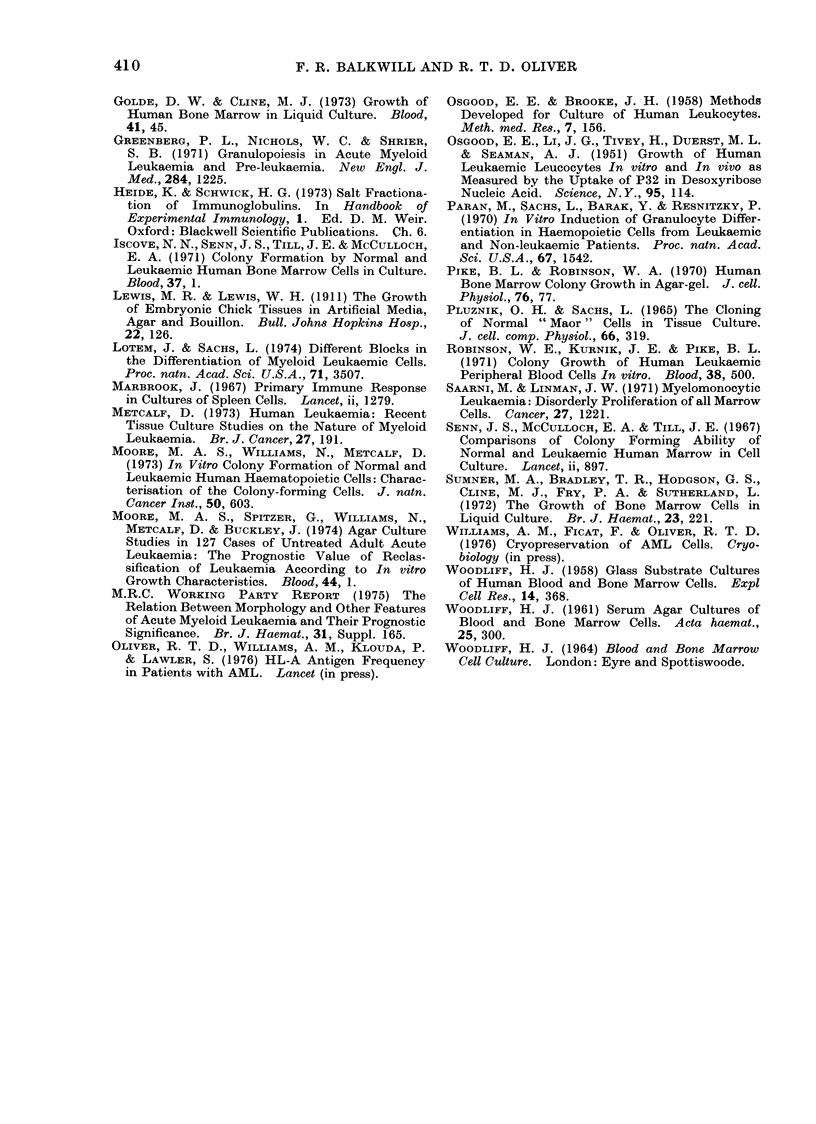# Diagnostic and prognostic significance of peripheral blood cultural characteristics in adult acute leukaemia.

**DOI:** 10.1038/bjc.1976.65

**Published:** 1976-04

**Authors:** F. R. Balkwill, R. T. Oliver

## Abstract

**Images:**


*Br. J. Cancer* (1976) **33**, 400

### DIAGNOSTIC AND PROGNOSTIC SIGNIFICANCE OF PERIPHERAL BLOOD CULTURAL CHARACTERISTICS IN ADULT ACUTE LEUKAEMIA

F. R. BALKWILL AND R. T. D. OLIVER

*From the Imperial Cancer Research Fund Department of Medical Oncology, St Bartholomew's Hospital, London E.C.*1

Received 26 November 1975 Accepted 2 December 1975

**Summary.—A simple liquid culture technique has been used to study peripheral blood from patients with acute myelogenous leukaemia. Evidence is presented that cells from morphologically identical types of leukaemia have differing capacity for " differentiation " from free floating blast cells into plastic-adherent, phagocytic, trypsin-resistant macrophage-like cells with Fc and $C_3$ receptors. Preliminary analysis suggests that patients whose cells have the greatest capacity for " differentiation " have a better chance of achieving complete remission.**

UNCONTROLLED proliferation of primitive cells and failure of normal differentiation are thought to be basic defects leading to acute leukaemia. The use of *in vitro* culture is an obvious way to investigate this problem, but until the early 1960s the majority of research workers found the culture of normal and abnormal haemopoietic cells to be very difficult (Woodliff, 1964). In a variety of systems (Woodliff, 1964; Woodliff, 1958; Woodliff, 1961; Osgood and Brooke, 1958; Lewis and Lewis, 1911; Carrel and Burrows, 1911) cells only survived a few days and showed little evidence of multiplication or differentiation.

With the discovery of growth stimulating factors such as CSF (colony stimulating factor) and the introduction of a semisolid agar culture system, actual growth and differentiation of mouse (Pluznik and Sachs, 1965; Bradley and Metcalf, 1966) and human (Pike and Robinson, 1970) bone marrow cells were obtained.

Despite some reports to the contrary (Senn, McCulloch and Till, 1967; Iscove *et al.*, 1971) the agar system has been found to support the growth of acute and chronic granulocytic leukaemia cells (Paran *et al.*, 1970; Moore, Williams and Metcalf, 1973; Robinson, Kurnik and Pike, 1971). Variation between reports is considerable (Senn *et al.*, 1967; Paran *et al.*, 1970; Moore *et al.*, 1973; Robinson *et al.*, 1971; Brown and Carbonne, 1971; Greenberg, Nichols and Shrier, 1971; Iscove *et al.*, 1971), but this may be due to differences in method and the small number of patients in each study. In a study of 127 cases of pre-treatment acute leukaemias, Moore *et al.* (1974) reported a variety of growth patterns indicating a spectrum of normal to abnormal growth and differentiation. Although agar cultures have contributed much to our knowledge of normal and abnormal myelopoiesis and factors influencing it (Metcalfe, 1973), cellular mobility and interaction are not easily studied using this system and retrieval of cells is difficult. Therefore, it was a welcome advance when liquid culture systems for mouse (Sumner *et al.*, 1972) and human bone marrow cells (Golde and Cline, 1973) which supported both growth

Reprint requests to: Dr R. T. D. Oliver, Department of Medical Oncology, St. Bartholomew's Hospital, London, E.C.1.

and differentiation were reported. Using Marbrook diffusion chambers (Marbrook, 1967) Golde and Cline (1973) reported growth of normal human bone marrow and some leukaemic cells and showed that these cells were capable of growth in the absence of an external source of CSF. However, the complexity of the apparatus required limited the generalized use of this technique.

Other workers have reported leukaemia cell growth in less complicated culture with, and in some cases, without stimulating factors (Aye, Till and McCulloch, 1972; Aye *et al.*, 1974*a*; Aye, Till and McCulloch, 1974*b*), but again only small numbers of patients were studied and some of the patients had recently received chemotherapy. These workers found no evidence of cellular differentiation (Aye *et al.*, 1974*a*).

We reported in 1974 that in a simple quantitative liquid microculture system the majority of AML cells would proliferate (Balkwill, Pindar and Crowther, 1974) and in this paper we wish to describe morphological and functional studies on these cells grown in both the microsystem and larger culture vessels.

### MATERIALS AND METHODS

*Preparation of the cells.*—Fresh peripheral blasts from untreated patients with AML, or buffy-coat cells from normal individuals, were separated from heparinized blood (300 units mucous preservative-free heparin to 10 ml blood) by the addition of 1% methyl cellulose (3 ml to 10 ml blood). This was allowed to sediment for 15 min at room temperature, when the buffy coat was removed and washed once in medium RPMI 1640 (Gibco Biocult Ltd). Liquid-nitrogen-stored pre-treatment peripheral blasts were thawed at 37 °C and then diluted dropwise over 10 minutes with RPMI 1640 to ensure good viability as previously described (Balkwill *et al.*, 1974; Williams, Ficat and Oliver, 1975). These cells were washed once.

*Culture of the cells.*—For culture, cells were re-suspended at $1 \times 10^6$/ml in medium 199 with antibiotics (Wellcome Reagents Ltd) supplemented by L-asparagine 0·2 mg/ml, extra L-glutamine (1 ml 200 mM solution/100 ml medium) (Flow Laboratories Ltd) and 10% Foetal Bovine serum, Batch No. 417095 (Flow Laboratories Ltd). Ten ml aliquots of the cell suspension were added to tissue culture flasks (Falcon Plastics 3013) or 0·2 ml aliquots added to Falcon 3040 microtitre plates. Sealed tissue culture flasks and plates with lids were incubated in 5% $CO_2$ 85% humidity atmosphere at 37 °C.

*Observation and Photomicroscopy.*—Observation and photomicroscopy of living cells were carried out under phase contrast microscopy using a Wild M40 inverted microscope equipped with a 35 mm camera attachment and using Agfapan 25 film. Fixed and stained cells were examined and photographed on either a Wild M20 microscope or a Leitz Ortholux 1 Microscope using Kodak photomicrograph 2483 film.

*Observation of stained cells.*—Non-adherent cells were smeared on glass slides or cytocentrifuge preparations made. Adherent monolayers were washed ×3 in Medium 199 and then fixed in methanol, stained *in situ* and then the sides and top of the bottle removed with pliers. The stained monolayers were mounted with Ceedol (Raymond Lamb) or neutral glycerin jelly (G. T. Gurr Ltd) as conventional slide mounts degraded the plastic.

*Assessment of the uptake of tritiated thymidine.*—The cells grown in microculture were pulsed for 16 h with $H^3$-thymidine sp. act. 5 Ci/mmol (Amersham Radiochemicals) to give a final concentration in the wells of 0·5 $\mu$Ci/ml. The cultures were harvested using a cell harvesting machine, the filter papers dried and counted in an Intertechnique ABAC SL40 Liquid Scintillation counter. Values obtained from 6 wells were averaged for each estimation.

*Phagocytosis.*—Cultures of *Staph. albus* grown overnight in nutrient broth were washed ×2 in PBS and then resuspended in RPMI 1640 + 10% human AB serum. These were incubated at 37 °C for 30 min and then washed ×2 again before being added to the cells in the culture flasks. The cultures were incubated at 37 °C for two hours and then the non-adherent cells + bacteria were removed from the bottles, washed ×3 with slow spinning in order to remove the majority of non-ingested bacteria, and then cytocentrifuge preparations were made of the cell pellet. Adherent cells were washed ×3 in RPMI 1640 by gentle pipetting and then wet-fixed in methanol. All preparations were Gram stained and counterstained with neutral red.

*Trypsin resistance of adherent cells.*—Non-adherent cells were removed from the cultures and the remaining adherent cells washed ×3 in RPMI 1640. The number of adherent cells in 8 marked fields on each bottle was then counted and Medium 199 containing 0·1% trypsin added for 10 min at 37 °C. The monolayers were washed ×3 in Medium 199 and the remaining cells counted in the same fields. The percentage cell reduction due to the presence of trypsin was calculated.

*Receptor techniques.*—*E* Sheep red blood cells (SRBC) preserved in Alsever's solution (Wellcome Reagents Ltd) were washed ×3 in medium before use.

*EA* 1/100 dilution of a rabbit anti-sheep red cell serum (Wellcome Reagents Ltd) was added to an equal volume of 5% SRBC in Medium 199 and incubated 30 min at 37 °C. The coated SRBC were washed ×3 in Medium 199 and stored at 4 °C until used (not more than 4 h after making up).

*E-IgM and EAC* Rabbit anti-SRBC IgM fraction was prepared by i.v. injection of SRBC washed ×2 in PBS. 0·5 ml of a 10% solution was injected and 7 days later serum collected. The euglobulin fraction of this serum was precipitated by ammonium sulphate (Heide and Schwick, 1973) and dialysed against Tris/HCl buffer pH 8·2 for one day. Column fractionation of a Sephadex G 200 column resulted in a single peak which was concentrated by an Amicon Diaflow Filter P30 (Amicon Ltd) and stored at −179 °C until needed. A 1/100 dilution of this fraction in Medium 199 was added to an equal volume of a 5% SRBC suspension, E, and incubated 30 min at 37 °C. This E-IgM complex was washed ×3 in Medium 199. The source of complement was human AB serum stored at −179 °C in small aliquots. This was thawed immediately before use and equal volumes of a 1/100 dilution of this and the E-IgM were incubated at 37 °C for 15 min. Both the E-IgM and the complement were suspended in CFT buffer with 0·15 mM $CaCl_2$ and 0·5 mM $MgCl_2.6H_2O$. After washing ×3 in Medium 199 the E-IgM and the EAC were stored at 4 °C until used (not more than 4 h after making up).

The microculture system was used for the evaluation of receptors, thus allowing only small volumes of reagents to be used and each test done in duplicate. The non-adherent cells were removed from the wells, washed ×2 and then $2 \times 10^5$ of these added to a 2% solution of E, E-IgM, EA(IgM)C in Beckman tubes. The Beckman tubes were then incubated at 37 °C with a rotation of 22 rev/min for 30 min and then allowed to settle. Rosettes were counted using a Wild M10 microscope under phase contrast and at least 400 cells on two different haemocytometer preps, counted for each parameter.

After removal of the non-adherent cells, the adherent cells were washed ×3 by gentle pipetting in Medium 199 and then 2% solution of the above erythrocyte and antibody reagents added to each well. After 30 min incubation at 37 °C, unattached SRBC were removed by immersion of the plate several times into a bath of warm medium. Each parameter was assessed using a Wild M40 inverted microscope on duplicate wells with at least 400 cells being counted. All rosettes were scored as the attachment of five or more SRBC/cell.

### RESULTS

*Microculture system.*—Peripheral blood (PB) taken at the time of diagnosis from 48 out of 58 (83%) patients with AML grew in microculture with an incorporation from 1000 to 50,000 ct/min/well of $^3$H-thymidine measured after 4 days of culture.

*Macroculture—morphological observations.*—In 12/60 (20%) successfully grown flask cultures from acute myelogenous leukaemia pretreatment peripheral blood, free-floating rounded cells persisted throughout the period of culture, but in the remaining 80% during the period of culture, elongated plastic-adherent cells developed (Fig. 1). The free-floating cells were found to survive up to 28 days in culture with frequent mitoses being observed, whereas the adherent cells survived up to 6 months, often forming multinucleate cells and showing no evidence of mitoses.

FIG. 1.—Development of a leukaemia cell culture into adherent and non-adherent cells. ×150.

FIG. 2.—May–Grünwald–Giemsa stained cytocentrifuge preparations of supernatant populations from leukaemic peripheral blood after 7 days culture showing mainly undifferentiated cells. ×135.

May–Grünwald–Giemsa (MGG) stained preparations of the non-adherent cells revealed a large variety and spectrum of normal and abnormal differentiation of monocyte and granulocytes, precursor cells (Fig. 2) and in some cultures a few mature polymorphs (Fig. 3). The adherent population, however, had the appearance of mature mononuclear phagocytes (see Fig. 1).

In order to investigate the relevance of these two populations of cells to the disease, each pre-treatment fresh PB culture was classified as to the number of typical adherent cells per $\times 150$ high power phase contrast field. The cultures were always scored on the 3rd or 4th day of growth. The cultures were classified as shown in Fig. 4. +++ cultures >50, ++ cultures 20–50, + cultures 1–20, − cultures <1 fusiform cell per high power field.

*Functional studies.*—The liquid nitrogen stored cells when cultured showed the same characteristic morphology as cells that had not been frozen, and these have been used for the detailed functional studies described below. All cell preparations had greater than 95% viability assessed by trypan blue dye exclusion.

*Phagocytosis.*—The adherent cells were found to be avidly phagocytic for the opsonized *Staph. albus* particles (Fig. 5) whereas the majority of non-adherent cells were poorly phagocytic. Six leukaemias and one normal were studied and the results shown in Table I.

*Trypsin resistance.*—The results in Table II indicate the percentage of plastic-adherent cells which are trypsin-resistant in +++ and + cultures and compares this with normal peripheral blood cultures and human embryo fibroblasts. It is clear that in the +++

FIG. 3.—May–Grünwald–Giemsa stained cytocentrifuge preparations of supernatant populations from leukaemic peripheral blood after 7 days culture showing some myeloid and monocytoid differentiation. $\times 135$.

FIG. 4.—Different types of leukaemia peripheral blood cultures. ×150.

FIG. 5. Phagocytosis of *Staph. albus* by adherent cells from 4-day leukaemia cell culture. ×135.

TABLE I.—*Phagocytosis Experiments on Cells from Patients with AML*

| Patient no. | Classification of cell culture | Day 1 Adherent cells | Day 1 Supernatant cells | Day 4 Adherent cells | Day 4 Supernatant cells |
|---|---|---|---|---|---|
| 1 | +++ | 57* | 63 | 80 | 36 |
| 2 | +++ | 95 | 24 | 97 | 18 |
| 3 | +++ | 44 | NT | 100 | 19 |
| 4 | +++ | 40 | NT | 98 | 32 |
| 5 | + | 5 | 3 | 48 | 16 |
| 6 | — | No cells | 4 | No cells | 3 |
| | Normal control | 83 | 32 | 98 | NT |

NT.—Not tested.
* Percentage of cells containing ingested *Staph. albus*.
For explanation of classification see Fig. 4.

TABLE II.—*Trypsin Resistance Experiment on Cells from Patients with AML*

| Patient no. | Classification of cell culture | Percentage trypsin-resistant adherent cells |
|---|---|---|
| 1 | +++ | 89 |
| 2 | +++ | 79 |
| 3 | +++ | 88 |
| 4 | +++ | 84 |
| 5 | + | 44 |
| 6 | + | 38 |
| 7 | + | 13 |
| | Normal control | 87 |
| | Human embryo fibroblasts | Less than 1 |

For explanation of classification see Fig. 4.

cultures, plastic-adherent cells are as resistant to trypsin as normal peripheral blood macrophages, while the majority of plastic-adherent cells in + cultures are removed by trypsin treatment.

*Receptors.*—Eleven liquid nitrogen stored blast cell cultures (3 +++, 6 +, 2 —) and 3 fresh normal cell cultures were set up as described in *Methods* to assess the presence of the Fc and $C_3$ receptor on these cells pre-culture, and Days 1, 4–5 and 7–8 of culture. After culture a high proportion of the adherent cells were found to have both $C_3$ and Fc receptors as Fig. 6 shows. The finding that the EA rosettes were invariably phagocytosed during the incubation time, whereas the EAC rosettes were never ingested, was unequivocal throughout the whole series of experiments. Also, the value of E or E-IgM rosettes was never greater than 1%.

A summary of the results obtained in the series of receptor experiments is shown in Fig. 7. These results clearly show that mature cells possessing the $C_3$ and Fc receptor first developed in the supernatants of the +++ cultures, and then increasing numbers were found amongst the plastic-adherent cells. No differentiation of this kind was found in the +, — or the normal cultures studied, although at 7 days a few receptor positive cells did appear in the floating population.

*Relationship of type of culture to diagnosis.*—Table III shows the relationship of type of culture to diagnosis and indicates that although there is a definite tendency towards a greater percentage of adherent cells in patients with acute myelomonocytic leukaemia, this does not always occur. In addition, many of the so-called acute myelogenous leukaemias (32%) have quite large proportions of adherent cells (the +++ and ++ groups).

*Cultural characteristics and response to treatment.*—The patients in this study received standard chemotherapy induction using protocols which have been reported in detail elsewhere (Crowther *et al.*, 1973). Table IV shows the incidence of complete remission achieved in these patients relative to the cultural type. Fifty-four per cent of patients in the +++ and ++ group, 18% of the + and — group and 15% in the no growth

FIG. 6.—The demonstration of $F_c$ and $C_3$ receptors on adherent cells from 7-day leukaemia cell cultures. ×150.

TABLE III.—*Relationship of Cultural Characteristics to Haematological Diagnosis*

| | Myeloblastic | Myelomonocytic |
|---|---|---|
| +++ Stickers | 1 | 9 |
| ++ Stickers | 15 | 6 |
| + Stickers | 13 | 4 |
| − Non-stickers | 11 | 1 |
| No growth | 10 | 0 |

For explanation of classification see Fig. 4.

group achieved complete remission ($P < 0{\cdot}025$).

### DISCUSSION

The studies we have so far carried out on the cultural characteristics of acute myelogenous leukaemia cells in the peripheral blood have confirmed, firstly that these cells will proliferate actively *in vitro* without any external growth stimulating factors, as has been

FIG. 7.—Evolution of receptors in cell cultures.

TABLE IV

| Cultural characteristics | No. of patients | Achievement of complete remission (%) |
|---|---|---|
| + + + and + + | 24 | 54 |
| + and − | 27 | 18 |
| No growth | 7 | 15 |

$P < 0 \cdot 025$.

found by other workers (Golde and Cline, 1973; Aye *et al.*, 1974*a* and *b*; Osgood *et al.*, 1951). This *in vitro* proliferation is not found in normal peripheral blood. Secondly, the studies have indicated that cells from some patients are capable of differentiating along the monocyte/macrophage pathway, developing phagocytic capacity, trypsin resistance and Fc and $C_3$ receptors. Similar differing degrees of differentiation have been reported from studies of murine myeloid leukaemia (Lotem and Sachs, 1974).

This raises again the problem of the true cell of origin of myeloid leukaemia and what is the difference between acute myeloblastic and myelomonocytic leukaemia. Our findings would suggest that a high percentage of morphologically myeloblastic leukaemias have abnormalities of the monocytic-macrophage series. This finding is supported by the electron microscopy studies of Glick and Horn (1974) who found the majority of AMLs to have an excess of monocyte precursors in their bone marrow, and the results from Saarni and Linman (1971) who, in a series of 381 AMLs, showed involvement of more than one cell line in 38% of patients.

The finding that cultural characteristics significantly influence the prognosis of acute myelogenous leukaemia may be somewhat surprising to many workers, especially as very few prognostic features have been found in this disease. The MRC Working Party Report (1975) on 272 cases found that initial morphological differentiation had no prognostic significance except in the small well-defined promyelocytic group. However, in 1974 Moore published a study of 127 cases of untreated acute leukaemia (Moore *et al.*, 1974) which had been cultured in semi-solid agar, and found cultural patterns of prognostic significance. Although our study represents a smaller number of cases, there is a suggestion that our technique is detecting the same group of patients as their good prognostic group (small-cluster-forming leukaemia) which had a higher percentage of monocytes than the poor prognosis group and occurred at approximately the same frequency.

The fact that cultural characteristics seem to influence prognosis raises the question of how these factors could work *in vivo*. The well-established link between anaplasia and bad prognosis has been shown in many solid tumours and our evidence that maturation *in vitro* seems to be the mechanism by which the adherent cells are produced may indicate that the leukaemias show the same association as do other cancers. It may be that the difference between the groups relates to the relative sensitivity to chemotherapeutic drugs applied during remission induction, though recent studies on HL-A antigens in these patients suggest that there may be genetic factors involved (Oliver *et al.*, 1976).

One further possibility is that these macrophages produced in culture may be normal ones produced in response to leukaemia. Eccles and Alexander (1975) have found a strong correlation between the presence of macrophage in animal tumours and a good prognosis, and Gauci, Wrathmell and Alexander (1975) investigating an experimental rat myelomonocytic leukaemia using a similar culture system to ours, have shown that macrophages produced in cultures of these cells were host-derived when the leukaemia was passaged in an $F_1$ Hybrid. However, their experiments could not totally exclude induction of leukaemia in the host cells. In the human leukaemias we have studied this seems to be an unlikely explanation as the macrophages develop over a period of one week from cells in the supernatant.

This work was supported by the Imperial Cancer Research Fund and initiated under the guidance of the late Professor Gordon Hamilton Fairley. We are grateful to Professor P. Alexander, Professor D. Crowther and Dr J. M. A. Whitehouse for helpful suggestions and criticisms and to Dr M. Waterfield for help with serum fractionation.

### REFERENCES

AYE, M. T., TILL, J. E. & MCCULLOCH, E. A. (1972) Growth of Leukaemia Cells in Culture. *Blood*, **40**, 806.

AYE, M. T., NIHO, Y., TILL, J. E. & MCCULLOCH, E. A. (1974*a*) Studies of Leukaemic Cell Population in Culture. *Blood*, **44**, 205.

AYE, M. T., TILL, J. E. & MCCULLOCH, E. A. (1974*b*) Interacting Populations Affecting Proliferation of Leukaemia Cells in Culture. *Blood* **45**, 205.

BALKWILL, F. R., PINDAR, A. & CROWTHER, D. (1974) Factors Influencing Microculture of Leukaemic Cells. *Nature, Lond.*, **251**, 741.

BRADLEY, T. R. & METCALF, D. (1966) The Growth of Mouse Bone Marrow Cells *In vitro*. *Aust. J. exp. Biol. med. Sci.*, **44**, 287.

BROWN, C. H. & CARBONNE, P. B. (1971) *In vitro* Growth of National and Leukaemic Bore Marrow. *J. natn. Cancer Inst.*, **46**, 989.

CARREL, A. & BURROWS, M. T. (1911) Culture de Moelle Osseuse et de Rate (Troisième Note). *C. r. Soc. Biol.*, **69**, 299.

CROWTHER, D., POWLES, R. L., BATEMAN, C. J. T., BEARD, M. E. J., GAUCI, C. L., WRIGLEY, P. F. M., MALPAS, J. S., HAMILTON FAIRLEY, G. & SIR RONALD BODLEY SCOTT (1973) Management of Adult Acute Myelogenous Leukaemia. *Br. med J.*, i, 131.

ECCLES, S. & ALEXANDER, P. (1975) Macrophage Content of Tumours in Relation to Metastatic Spread and Host Immune Reaction. *Nature, Lond.*, **250**, 667.

GAUCI, C. L., WRATHMELL, A. & ALEXANDER, P. (1975) The Origin and Role of Blood Borne Monocytes in Rats with a Transplantable Acute Myelogenous Leukaemia. *Cancer Letters*, **1**, 33.

GLICK, A. D. & HORN, R. G. (1974) Identification of Promonocytes and Monocytoid Precursors in Acute Leukaemia of Adults: Ultrastructural Cytochemical Observations. *Br. J. Haemat.*, **26**, 395.

GOLDE, D. W. & CLINE, M. J. (1973) Growth of Human Bone Marrow in Liquid Culture. *Blood*, **41**, 45.

GREENBERG, P. L., NICHOLS, W. C. & SHRIER, S. B. (1971) Granulopoiesis in Acute Myeloid Leukaemia and Pre-leukaemia. *New Engl. J. Med.*, **284**, 1225.

HEIDE, K. & SCHWICK, H. G. (1973) Salt Fractionation of Immunoglobulins. In *Handbook of Experimental Immunology*, **1**. Ed. D. M. Weir. Oxford: Blackwell Scientific Publications. Ch. 6.

ISCOVE, N. N., SENN, J. S., TILL, J. E. & MCCULLOCH, E. A. (1971) Colony Formation by Normal and Leukaemic Human Bone Marrow Cells in Culture. *Blood*, **37**, 1.

LEWIS, M. R. & LEWIS, W. H. (1911) The Growth of Embryonic Chick Tissues in Artificial Media, Agar and Bouillon. *Bull. Johns Hopkins Hosp.*, **22**, 126.

LOTEM, J. & SACHS, L. (1974) Different Blocks in the Differentiation of Myeloid Leukaemic Cells. *Proc. natn. Acad. Sci. U.S.A.*, **71**, 3507.

MARBROOK, J. (1967) Primary Immune Response in Cultures of Spleen Cells. *Lancet*, ii, 1279.

METCALF, D. (1973) Human Leukaemia: Recent Tissue Culture Studies on the Nature of Myeloid Leukaemia. *Br. J. Cancer*, **27**, 191.

MOORE, M. A. S., WILLIAMS, N., METCALF, D. (1973) *In Vitro* Colony Formation of Normal and Leukaemic Human Haematopoietic Cells: Characterisation of the Colony-forming Cells. *J. natn. Cancer Inst.*, **50**, 603.

MOORE, M. A. S., SPITZER, G., WILLIAMS, N., METCALF, D. & BUCKLEY, J. (1974) Agar Culture Studies in 127 Cases of Untreated Adult Acute Leukaemia: The Prognostic Value of Reclassification of Leukaemia According to *In vitro* Growth Characteristics. *Blood*, **44**, 1.

M.R.C. WORKING PARTY REPORT (1975) The Relation Between Morphology and Other Features of Acute Myeloid Leukaemia and Their Prognostic Significance. *Br. J. Haemat.*, **31**, Suppl. 165.

OLIVER, R. T. D., WILLIAMS, A. M., KLOUDA, P. & LAWLER, S. (1976) HL-A Antigen Frequency in Patients with AML. *Lancet* (in press).

OSGOOD, E. E. & BROOKE, J. H. (1958) Methods Developed for Culture of Human Leukocytes. *Meth. med. Res.*, **7**, 156.

OSGOOD, E. E., LI, J. G., TIVEY, H., DUERST, M. L. & SEAMAN, A. J. (1951) Growth of Human Leukaemic Leucocytes *In vitro* and *In vivo* as Measured by the Uptake of P32 in Desoxyribose Nucleic Acid. *Science, N.Y.*, **95**, 114.

PARAN, M., SACHS, L., BARAK, Y. & RESNITZKY, P. (1970) *In Vitro* Induction of Granulocyte Differentiation in Haemopoietic Cells from Leukaemic and Non-leukaemic Patients. *Proc. natn. Acad. Sci. U.S.A.*, **67**, 1542.

PIKE, B. L. & ROBINSON, W. A. (1970) Human Bone Marrow Colony Growth in Agar-gel. *J. cell. Physiol.*, **76**, 77.

PLUZNIK, O. H. & SACHS, L. (1965) The Cloning of Normal " Maor " Cells in Tissue Culture. *J. cell. comp. Physiol.*, **66**, 319.

ROBINSON, W. E., KURNIK, J. E. & PIKE, B. L. (1971) Colony Growth of Human Leukaemic Peripheral Blood Cells *In vitro*. *Blood*, **38**, 500.

SAARNI, M. & LINMAN, J. W. (1971) Myelomonocytic Leukaemia: Disorderly Proliferation of all Marrow Cells. *Cancer*, **27**, 1221.

SENN, J. S., MCCULLOCH, E. A. & TILL, J. E. (1967) Comparisons of Colony Forming Ability of Normal and Leukaemic Human Marrow in Cell Culture. *Lancet*, ii, 897.

SUMNER, M. A., BRADLEY, T. R., HODGSON, G. S., CLINE, M. J., FRY, P. A. & SUTHERLAND, L. (1972) The Growth of Bone Marrow Cells in Liquid Culture. *Br. J. Haemat.*, **23**, 221.

WILLIAMS, A. M., FICAT, F. & OLIVER, R. T. D. (1976) Cryopreservation of AML Cells. *Cryobiology* (in press).

WOODLIFF, H. J. (1958) Glass Substrate Cultures of Human Blood and Bone Marrow Cells. *Expl Cell Res.*, **14**, 368.

WOODLIFF, H. J. (1961) Serum Agar Cultures of Blood and Bone Marrow Cells. *Acta haemat.*, **25**, 300.

WOODLIFF, H. J. (1964) *Blood and Bone Marrow Cell Culture*. London: Eyre and Spottiswoode.